# The mental health impact of repeated COVID-19 enforced lockdowns in England: evidence from the UK Household Longitudinal Study

**DOI:** 10.1192/bjo.2024.803

**Published:** 2025-01-13

**Authors:** Rashpal K. Dhensa-Kahlon, Siu Tim Wan, Jacqueline A.-M. Coyle-Shapiro, Kevin Rui-Han Teoh

**Affiliations:** School of Psychological Sciences, Birkbeck, University of London, London, UK; Aston Medical School, Aston University, Birmingham, UK; Department of Management, London School of Economics & Political Science, London, UK; and Department of Management, California State University, San Bernardino, California, USA; Birkbeck Business School, Birkbeck, University of London, London, UK

**Keywords:** COVID-19, lockdowns, mental health, England, UKHLS

## Abstract

**Background:**

Research shows initial COVID-19 lockdowns increased population mental distress. Yet, the mental health impact of repeated lockdowns in England remains unknown.

**Aims:**

To: (a) explore changes in population mental health symptoms over the COVID-19 pandemic period (March 2020 to March 2021) in England, comparing this with trends from a decade before (2009–2019) as well as after (2021–2023); (b) compare the mental health impact of each of the three lockdowns in England with periods of eased restrictions, determining who was most affected; (c) examine the impact of demographics and distinct time periods on the prevalence of mental health symptoms.

**Method:**

A secondary analysis of a national longitudinal cohort study, utilising data from Waves 1–13 of the UK Household Longitudinal Study and from Waves 1–9 of the COVID-19 Survey. Mental health was assessed using the 12-item General Health Questionnaire. Student *t*-tests and logistical regressions were conducted.

**Results:**

There was a significant increase in the prevalence of self-reported symptoms of mental health during England's pandemic period, encompassing three lockdowns, compared with the average of rates from 10 years before. Rates of reported mental health symptoms were not significantly different across each lockdown, but were significantly higher than pre-pandemic rates, declining with eased restrictions. Rates from the end of lockdown to May 2023 revealed elevated mental health symptoms compared with pre-pandemic. Elevated symptoms were observed for women, people homeworking, those with health conditions, individuals aged 30–45 years and those experiencing loneliness.

**Conclusion:**

Repeated lockdowns in England had a substantial impact on mental health, indicating requirements for ongoing mental health support.

A plethora of research conducted early in the wake of the COVID-19 pandemic explored the psychological effects of initial lockdowns in 2020 worldwide, showing that lockdown conditions were associated with increased mental health symptoms in populations.^1–5^ Whereas the rationale underlying lockdown was clear, questions remain unanswered about the mental health impact of *repeated* lockdowns (i.e. beyond the first 6 months of the pandemic), such as those imposed on England three times between April 2020 and March 2021. There is still much to learn from the pandemic's effect on mental health, and specifically, about the mental health impact of repeated lockdowns which has evaded research interest, and yet is vital in aiding health service providers and policymakers with planning for future health emergencies, and for supporting vulnerable groups for whom mental health symptoms likely persisted beyond the pandemic's official end.^6,7^

## Initial COVID-19 lockdowns and mental health

Following the rapid spread of COVID-19, in early 2020, governments worldwide issued stay-at-home orders to limit the spread of this virus. By April 2020, over 100 countries had instigated lockdown. Existing research on the mental health impact of initial lockdowns and the period immediately after showed marked increases in people reporting symptoms of psychological distress, including in the UK,^2,3,5,8,9^ Switzerland,^10^ Germany,^11^ Italy,^12^ the USA,^8,13^ China, Turkey, Denmark and Nepal.^14^ As restrictions were eased, available evidence across the globe painted a mixed picture. For example, in the UK, there appeared to be a prolonged deterioration in mental health such that symptoms of mental distress persisted beyond the end of the first lockdown,^1,8^ and although there were some improvements in overall population mental health by October 2020, these were nonetheless markedly higher than pre-pandemic levels.^1,15^ On the other hand, data from the USA found that while mental health problems rose early during the COVID-19 pandemic, there were improvements to population mental health towards the end of lockdown^16^ with rates returning to those similar to pre-pandemic levels by June 2020.^8^

## Repeated COVID-19 lockdowns and mental health

In response to the rising spread of new variants, subsequent lockdowns were ordered in many parts of the world, subjecting populations to ongoing impositions on movement for prolonged periods. However, few studies have investigated population mental health beyond the first 6 months of the pandemic, with existing research globally evidencing diverse findings. Findings from a micro-economic study^17^ showed that women reported much higher levels of anxiety and depression than males during the UKs pandemic period encompassing three lockdowns. A study during emergency declarations in Japan found that although mental health symptoms decreased during two lockdowns overall, cumulative negative effects of social isolation and loneliness affected young adults and those reporting high levels of loneliness.^18^ Similarly, evidence from three lockdowns in Australia found that while there was not a deterioration of mental health over time, more adverse mental health symptoms were experienced by younger people, individuals with caring responsibilities and people with prior mental health problems.^19^ These studies drew on convenience samples and are limited by excluding a lack of comparable pre- (baseline) or post-pandemic data against which to measure changes in rates of mental health.

Taken together, existing evidence illuminates two possibilities. On one hand, people may have habituated to lockdown and living with adversity, showing psychological adaption to the pandemic. Elevated mental health symptoms may therefore be higher during lockdown, but transient, returning to more stable levels with eased restrictions. On the other hand, repeated lockdowns may have enduring effects on mental health that persist beyond eased restrictions. Research indicates that the mental health of some population sub-groups was disproportionately negatively affected. Women, young adults, people with existing health conditions and those from Black, asian and minority ethnic backgrounds were more at risk than men, older adults, people without existing health conditions and those from white backgrounds.^1,4,5,8,20^

Three additional factors merit investigation. First, for those working from home, lockdown repeatedly disrupted work patterns through the reconfiguration of remote work or navigating paid work alongside caregiving demands,^21^ blurring the lines between work and family domains. This might have impaired mental health or improved it, given the availability of more time for family and reduced commuting.^22,23^ Second, loneliness, a major public health concern, has emerged as a consequence of the pandemic and lockdown specifically,^6,24^ with evidence from early in the pandemic showing that people with existing mental health diagnoses, younger adults, women and students at greater risk of experiencing high levels of loneliness.^25^ Considering its association with anxiety and depression,^26,27^ loneliness may pose a risk for increased mental health problems over repeated lockdowns. Third, for individuals with pre-existing health conditions,^6^ lockdown may have exacerbated symptoms through the loss of access to health support or positive activities.

## Aims

Much previous research is limited by relying on cross-sectional analysis of data with small convenience samples, using unvalidated measures of mental health, and has not drawn on data from before the pandemic, thereby reducing the generalisability of findings. We drew on data from a national longitudinal cohort study which provided access to a nationally representative sample of UK adults, allowing an assessment of changes in mental health for population subgroups, as well as a validated measure of mental health. Governments of the four nations of the UK responded to the pandemic with a range of measures and diverged in the implementation of lockdown^1^. We therefore focused our analysis on England to provide a more nuanced insight into patterns of mental health across demographics. The present research had three aims. First, to explore changes in the prevalence of elevated mental health symptoms over England's COVID-19 pandemic period (March 2020 to March 2021), comparing this with trends from the decade before (‘pre-pandemic’, January 2009 to January 2019) and period after (‘post-pandemic’, January 2021 to May 2023) to identify patterns in population mental health symptoms triggered by a public health emergency. Second, to examine the mental health impact of each of the three lockdowns in England as well as when restrictions were eased (Lockdown-1: 23 March to 1 June 2020; Lockdown-2: 2 November to 2 December 2020; Lockdown-3: 4 January to 29 March 2021; see Supplementary Appendix 1 for details) to determine who was most affected (by gender, ethnicity, age, loneliness, work status and long-term health conditions). Third, to investigate the impact of demographic factors and distinct time periods (lockdown/non-lockdown) on the prevalence of elevated mental health symptoms.

## Method

### Study design and sample

We conducted a secondary longitudinal analysis using two data-sets. The first was the UK Household Longitudinal Study (UKHLS; a nationally representative ongoing panel survey on the economic conditions, health, and well-being of more than 40 000 UK households^28,29^ that began in 2009. The survey is based on a clustered-stratified probability sample. Each data collection wave of the UKHLS is conducted over 2 years, with participants interviewed online, face to face or via telephone surveys. Second, we utilised data from the monthly COVID-19 web survey launched by the UKHLS following the onset of the pandemic, with all eligible household members aged 16 or older in April 2020 invited to take part.^30,31^

Understanding Society is based at the Institute for Social and Economic Research at the University of Essex. Research data are shared by the UK Data Service. The University of Essex Ethics Committee has approved all data collection for the Understanding Society main study and COVID-19 wave. The study was additionally approved by a University Committee (Ref: OPEA-22/23-08).

We utilised data for participants in England aged 18 years and over who completed in full the 12-item General Health Questionnaire (GHQ-12) survey on each wave of the COVID-19 survey (Waves 1–9; *n* = 6093) and also participated in the surveys conducted by the UKHLS (Waves 1–13; *n* = 3424 to 6091). Participants’ responses were linked across waves using a household identifier. Missing data for a very small proportion of the sample (ranging from 0 to 1.03% participants across variables) was managed through listwise deletion. Data collection for the UKHLS was carried out annually from 1 January 2009, with most of the fieldwork taking place over the course of 2 years. Waves therefore overlap in their data collection periods (e.g. Wave 9: from 1 January 2017 to 31 May 2019; Wave 10 from 1 January 2018 to 31 May 2020). The COVID-19 survey started in April 2020, with Waves 2–4 taking place monthly (from May to July 2020), Waves 5–8 bi-monthly (September 2020 to March 2021) and Wave 9 in September 2021. Lockdown-1 is associated with Waves 1–3, Lockdown-2 with Wave 6 and Lockdown-3 with Waves 7–8. We refer to eased restrictions as non-lockdown. Non-Lockdown-1 is associated with Waves 4 and 5, with non-Lockdown-2 associated with Wave 9.

### Measures

#### Mental health

Self-reported mental health symptoms are measured in the UKHLS and the COVID-19 surveys using GHQ-12, a validated tool assessing non-psychotic psychiatric cases in the population. We utilised the entire set of GHQ assessments conducted from 2009 to December 2019 in the UKHLS data-set, and from April 2020 to September 2021 in the COVID-19 survey. In the GHQ-12, participants reported how they had been feeling over the last few weeks on 12 symptoms (such as difficulties with sleep, concentration, problems in decision-making and strain) on a 4-point scale, with a high score representing more frequent symptoms of mental health The UKHLS recoded the values of individual items on the GHQ-12 of 1 and 2 to 0, and values of 3 and 4 to 1 before summing them to produce a total ranging from 0 to 12. The cut-off for the threshold measure to detect elevated levels of mental health symptoms was a score of 3 or more, as validated in past research.^32,33^

Demographic variables were extracted on gender (male and female) and age (18–29, 30–45, 46–59, ≥60 years) from the UKHLS and full COVID-19 survey. We also extracted ethnicity (White, Mixed, Asian, Black, other), work location (home versus at employer), long-term health condition (health condition, no health condition) and loneliness (hardly ever or never, some of the time, often) levels in the previous 4 weeks from the full COVID-19 survey. Supplementary Table A available at https://doi.org/10.1192/bjo.2024.803 presents the demographic details of the sample. These variables reflect our chosen constructs consistently across all data waves.

### Data analysis

All analyses and graphs were carried out and produced in R (using RStudio) version 4.1 for Windows. We used population weights from the data-sets as per guidelines provided by Understanding Society.

To test our first aim, we conducted student *t*-tests to compare the changes in the prevalence of elevated mental health symptoms in the England population for people exceeding the cut-off threshold scores on the GHQ-12 (⩾3) between the decade before the pandemic (drawing on the UKHLS Waves 1–13 each year from 2009 to 2023), during the pandemic period (COVID-19 survey, Waves 1–9 from April 2020 to September 2021) and after the pandemic (UKHLS Wave 13 from January 2021 to May 2023). In calculating GHQ-12 scores we followed convention in calculating according to the UK financial year (April 1 to March 31) and grouped participants across waves. All missing values were given a negative value to indicate missing data.

For our second aim, we carried out further independent *t*-tests to compare the prevalence of elevated mental health symptoms over each of the three lockdowns with the non-lockdown periods (that followed Lockdown-1 and 3) when restrictions were eliminated. In examining rates of mental health symptoms during lockdown with periods of eased restrictions, available data permitted a comparison of Lockdown-1 with the ‘non-Lockdown-1’ period immediately after, and Lockdown-3 with the ‘non-Lockdown-2’ period immediately after. The student *t*-test was repeated for each demographic category.

Addressing our final aim, we performed a logistic regression analysis on the longitudinal data to examine the impact of various demographic factors at Wave 1 of the COVID-19 survey (such as gender, age group, ethnicity, long-term health conditions, working conditions and loneliness) and distinct time periods (including each lockdown and non-lockdown phase) on the prevalence of elevated mental health symptoms across all waves of the COVID-19 survey.

## Results

### Prevalence of elevated mental health symptoms in England: pre-pandemic (January 2009–January 2019), during lockdowns, and post-pandemic (January 2021–May 2023)

**Fig. 1:**
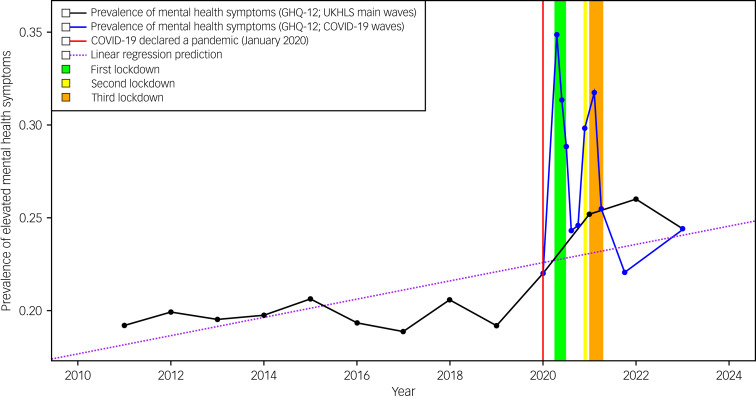
The prevalence of elevated mental health symptoms in England from January 2009 to May 2023 (as measured by the 12-item General Health Questionnaire).

The prevalence of elevated mental health symptoms in individuals in the 10 years before the pandemic, at 19.85% (95% CI 18.98–20.72), increased significantly during the pandemic on both the COVID-19 Survey (27.97% [95% CI 26.74–29.20]) and the UKHLS Survey (25.58% [95% CI 24.01–27.14]) (Table 1; Fig. 1). During Lockdown-1, the prevalence of elevated mental health symptoms increased significantly to 31.67% (95% CI 30.10–33.25), a 60% rise compared with the pre-pandemic period average over 10 years; for Lockdown-2, there was a 54% increase (29.82% [95% CI 27.94–31.72]), and for Lockdown-3, a 44% increase (28.64% [95% CI 27.10–30.18]). There was a significant difference in the prevalence of elevated mental health symptoms between the pre-pandemic period and the second non-lockdown period (22.05% [95% CI 20.59–23.51]) in England when all restrictions were eased, indicating a 10% rise in the prevalence of reported elevated mental health symptoms. Analysis of the period between the easing of all restrictions at the end of Lockdown-3 (approx. September 2021) until May 2023 (post-pandemic) highlights elevated mental health symptoms (24.39% [95% CI 22.49–26.29]) compared with pre-pandemic rates (19.85% [95% CI 18.98–20.72]), a 22% increase. This same post-pandemic period also had lower prevalence rates than Lockdown-3 (28.64% [95% CI 27.10–30.18]), higher rates than the second non-lockdown period (22.05% [95% CI 20.59–23.51]) and no difference across the entire pandemic (as reflected in the entire COVID-19 waves of data).

**Table 1:** Comparison of prevalence of elevated mental health symptoms in England between the pre-pandemic period (captured by the UK Household Longitudinal Study Waves 1–10) to the COVID-19 pandemic period (captured by the COVID-19 Survey Waves 1–9)

First period	Second period	Significance	d.f.	Mean difference	First period mean	Second period mean
MW1–10 (pre-pandemic 2009–2019)	CSW (during pandemic)	<0.0001	6091	−0.08	19.85% [CI 18.98–20.72]	27.97% [CI 26.74–29.20]
MW1–10 (pre-pandemic 2009–2019)	L1 (first lockdown)	<0.0001	5668	−0.12	19.85% [CI 18.98–20.72]	31.67% [CI 30.10–33.25]
MW1–10 (pre-pandemic 2009–2019)	L2 (second lockdown)	<0.0001	5668	−0.11	19.85% [CI 18.98–20.72]	30.72% [CI 28.92–32.40]
MW1–10 (pre-pandemic 2009–2019)	L3 (third lockdown)	<0.0001	5668	−0.09	19.85% [CI 18.98–20.72]	28.64% [CI 27.10–30.18]
MW1–10 (pre-pandemic 2009–2019)	NL2 (last COVID-19 survey Sep 2021)	<0.0029	6091	−0.02	19.85% [CI 18.98–20.72]	22.05% [CI 20.59–23.51]
MW13 (post-pandemic 2021–2023)	NL2 (last COVID-19 survey in Sep 21)	<0.019	6091	0.02	24.39% [CI 22.49–26.29]	22.05% [CI 20.59–23.51]
MW13 (post-pandemic 2021–2023)	L3 (third lockdown)	<0.0001	5266	−0.04	24.39% [CI 22.49–26.29]	28.64% [CI 27.10–30.18]
MW1–10 (pre-pandemic 2009–2019)	MW13 (post-pandemic 2021–2023)	<0.0001	4207	−0.05	19.85% [CI 18.98–20.72]	24.39% [CI 22.49–26.29]
MW1–10 (pre-pandemic 2009–2019)	MW11–12 (during pandemic)	<0.0001	4207	−0.06	19.85% [CI 18.98–20.72]	25.58% [CI 24.01–27.14]
MW11–12 (during pandemic)	MW13 (post-pandemic 2021–2023)	0.20	3424	0.01	25.58% [CI 24.01–27.14]	24.39% [CI 22.49–26.29]

MW1–10, UKHLS Main Waves 1 to 10, pre-pandemic, January 2009–January 2019; CSW, Covid-19 Survey Waves 1 to 9, April 2020–September 2021; L1, First lockdown, 23 March–1 June 2020; L2, Second lockdown, 2 November–2 December 2020; L3, Third lockdown, 4 January–29 March 2021; NL2, Non-Lockdown 2, 30 March 2021, Wave 9 from Covid Survey; MW13, UKHLS Main Wave Survey, post-pandemic, January 2021 – May 2023; MW11–12, UKHLS Main Waves, January 2019–May 2022.

### Changes in population mental health between lockdown and non-lockdown periods

**Fig. 2:**
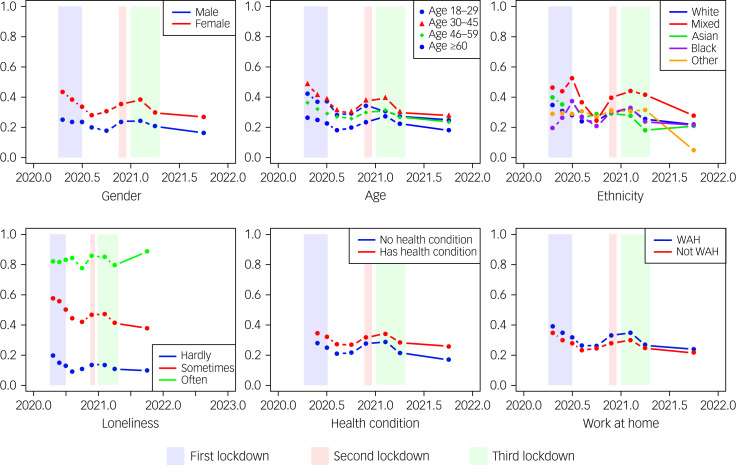
The prevalence of elevated mental health symptoms in England by subgroups (COVID-19 Survey Waves 1–9). Lines in each figure represent changes in mental health symptoms across demographics sub-groups over each of the COVID-19 survey waves, 2020–2021.

Table 2 (see also Fig. 2) presents changes in the prevalence of mental health symptoms reported across various demographic subgroups between collective lockdown and non-lockdown periods. During combined periods of England's three lockdowns, people reported elevated mental health symptoms (30.18% [95% CI 28.45–31.92]) compared with combined periods of non-lockdown (24.21% [95% CI 22.54–25.89]), with this difference representing a 25% increase in self-reported mental health symptoms during lockdowns. There are no significant differences between the rates of reported elevated mental health symptoms between each lockdown.

**Table 2:** Changes in the prevalence of elevated mental health symptoms in England between lockdown (COVID-19 Waves 1–3, 6–8) and non-lockdown (COVID-19 Waves 4, 5 and 9) periods

Category	Value	Significance	d.f.	Mean difference	Prevalence of symptoms of mental health during lockdown (%)	Prevalence of symptoms of mental health during non-lockdown (%)	Raw difference	Index difference (%)
Overall	Overall	<0.0001	2356	−0.06	30.18	24.21	5.97	24.65
Gender	Male	<0.0001	650	−0.06	24.29	18.43	5.86	31.79
	Female	<0.0001	1107	−0.06	35.76	29.38	6.38	21.71
Work location	Workplace	<0.0001	1230	−0.05	27.83	23.04	4.79	20.80
	Home	<0.0001	1335	−0.07	32.59	25.58	7.01	27.39
Age	18–29	0.0902	61	−0.05	30.09	25.44	4.65	18.27
	30–45	<0.0001	426	−0.07	35.32	28.05	7.27	25.91
	46–59	<0.0001	754	−0.05	27.57	22.31	5.26	23.58
	≥60	<0.0001	358	−0.06	26.87	20.78	6.10	29.34
Ethnicity	White	<0.0001	2125	−0.06	30.26	24.39	5.87	24.08
	Mixed	0.0612	4	−0.16	41.94	25.95	15.99	61.60
	Asian	0.7449	73	−0.02	23.63	21.70	1.93	8.90
	Black	0.0859	13	−0.16	25.88	10.13	15.74	155.34
	Other	0.3531	1	−0.1	45.42	34.95	10.47	29.95
Loneliness	Hardly	<0.0001	1814	−0.04	14.66	11.08	3.58	32.35
	Sometimes	<0.0001	956	−0.06	47.13	41.00	6.13	14.95
	Often	0.7469	117	0.01	83.76	84.85	−1.09	−1.29
Health condition	Previous health condition	<0.0001	1076	−0.06	26.22	19.79	6.43	32.47
	No previous health condition	<0.0001	842	−0.06	35.47	29.97	5.50	18.33

Index difference, percentage difference based on the first mean.

Notably, during lockdowns as compared with periods of non-lockdowns, women showed a larger increase in reported mental health symptoms than men, from 29.38% (95% CI 27.10–31.65) to 35.76% (95% CI 33.49–38.02) versus 18.43% (95% CI 15.90–20.97) to 24.29% (95% CI 21.61–26.98) respectively. However, the percentage rise observed was larger for men (31.80%) than women (21.75%). Increases in prevalence rates were higher during lockdown compared with non-lockdown for all age groups, except those aged 18–29 years which showed no difference. The following subgroups reported a significant increase in mental health symptoms between combined lockdown and non-lockdown periods: individuals who worked from home (compared with those who did not) – an approximate 27.4% increase, those from a White ethnicity – an approximate 24.1% increase, and people who reported feeling lonely hardly ever (a 32.31% increase) and sometimes (a 14.95% increase), as well as people with (a 18.35% increase) and without (a 32.49% increase) previous health conditions.

### Prevalence of elevated mental health symptoms during Lockdown-1 followed by subsequent easing of restrictions

During Lockdown-1 the prevalence of elevated mental health symptoms was at 31.02% (95% CI 28.73–33.32). Table 3 shows that elevated mental health symptoms were greater for women (36.34% [95% CI 33.58–39.11]) than men (25.27% [95% CI 21.70–28.83]), and for those aged 30–45 years (35.97% [95% CI 32.04–39.90]) than young adults (18–29 years; 31.55% [95% CI 23.50–39.60]). There were no significant differences to report between different ethnic groups. A higher proportion of people experiencing loneliness ‘often’ experienced greater elevated mental health symptoms (84.59% [95% CI 75.20–93.98]) compared with those feeling lonely sometimes (50.71% [95% CI 46.39–55.02]) or never (16.25% [95% CI 13.87–18.63]). Individuals working from home reported elevated mental health symptoms (33.56% [95% CI [30.81–36.36]) compared with those who did not (28.55% [95% CI 25.00–32.10]), a trend additionally observed in those with long-term health conditions (37.75% [95% CI 34.24–41.25]) than those without (26.14% [95% CI 23.53–28.75]).

**Table 3:** Population prevalence of elevated mental health symptoms during each lockdown and non-lockdown period by demographic subgroups

Demographic		L1 (%)	NL1 (%)	L2 (%)	L3 (%)	NL2 (%)
Overall	Overall	31.02	25.22	30.72	29.10	22.53
Gender	Male	25.27	19.76	24.21	23.40	16.23
	Female	36.34	30.10	36.99	34.54	28.16
Work location	Workplace	28.55	24.06	28.09	26.99	21.42
	Home	33.59	26.53	33.58	31.16	23.90
Age	18–29	31.55	27.59	32.00	27.74	22.08
	30–45	35.97	28.08	35.08	34.84	28.00
	46–59	27.77	23.38	29.83	26.22	20.50
	≥60	29.21	22.39	23.67	26.06	18.09
Ethnicity	White	30.95	25.30	30.92	29.25	22.88
	Mixed	49.27	26.97	35.45	38.66	24.17
	Asian	24.80	24.98	23.53	22.67	14.90
	Black	29.27	7.59	28.75	20.92	14.34
	Other	48.84	44.74	45.12	42.98	9.43
Loneliness	Hardly	16.25	11.23	14.81	12.88	10.83
	Sometimes	50.71	42.93	47.83	43.75	37.51
	Often	84.59	83.80	84.09	82.96	86.72
Health condition	Previous health condition	26.14	21.04	27.48	25.63	17.68
	No previous health condition	37.75	30.72	35.06	33.55	28.74

L1, Lockdown-1, 23 March–1 June 2020; L2, Lockdown-2, 2 November–2 December 2020; L3, Lockdown-3, 4 January–29 March 2021; NL1, Non-Lockdown-1, 1 June–1 November 2020; NL2, Non-Lockdown-2, 30 March 2021.

The prevalence of mental health symptoms when restrictions first eased (1 June to 1 November 2020) was at 25.22% (95% CI 23.00–27.45), representing a 20% decrease in reported mental health symptoms from the first lockdown period (Table 4). During this first non-lockdown period, a higher proportion of women (30.10% [95% CI 27.30–32.89]) reported mental health symptoms than men (19.76% [95% CI 16.70–22.82]; Table 3), as did adults aged 30–45 years (28.08% [95% CI 24.55–31.61]) than older adults aged 46–59 years (23.38% [95% CI 20.47–26.28]). Those with long-term health conditions were more likely to report mental health symptoms (30.72% [95% CI 27.54–33.91]) than those without (21.04% [95% CI 18.30–23.77]).

**Table 4:** Comparison of elevated mental health symptoms across each lockdown and no-lockdown period in England by demographic subgroups

Demographic		L1 *v.* NL1 (%)	L1 *v.* L2 (%)	L1 *v.* L3 (%)	NL1 *v.* L2 (%)	NL1 *v.* NL2 (%)	L2 *v.* L3 (%)	L3 *v.* NL2 (%)
Overall	Overall	−5.80***	−0.31	−1.93	5.49***	−2.70*	−1.62	−6.57***
Gender	Male	−5.51**	−1.06	−1.86	4.45**	−3.53*	−0.81	−7.17***
	Female	−6.25***	0.65	−1.81	6.89**	−1.93	−2.46	−6.37***
Work location	Workplace	−4.49*	−0.46	−1.56	4.03*	−2.64	−1.10	−5.57**
	Home	−7.06***	0.00	−2.42	7.06**	−2.62	−2.42	−7.26***
Age	18–29	−3.96	0.45	−3.81	4.41	−5.51	−4.26	−5.66
	30–45	−7.89***	−0.89	−1.13	7.00**	−0.08	−0.24	−6.84**
	46–59	−4.39**	2.05	−1.55	6.45***	−2.88	−3.60*	−5.72**
	≥60	−6.82*	−5.54	−3.16	1.28	−4.30*	2.39	−7.97***
Ethnicity	White	−5.65***	−0.03	−1.70	5.63***	−2.42*	−1.68	−6.37***
	Mixed	−22.30	−13.82	−10.61	8.48	−2.80	3.21	−14.49
	Asian	0.18	−1.27	−2.13	−1.45	−10.08	−0.86	−7.77
	Black	−21.68	−0.52	−8.34	21.16	6.75	−7.82	−6.58
	Other	−4.10	−3.72	−5.86	0.37	−35.31	−2.14	−33.55
Loneliness	Hardly	−5.02**	−1.43	−3.37*	3.58**	−0.40	−1.94	−2.04*
	Sometimes	−7.78**	−2.88	−6.95**	4.89*	−5.42*	−4.07	−6.25*
	Often	−0.79	−0.50	−1.62	0.29	2.92	−1.13	3.76
Health condition	Previous health condition	−5.10***	1.34	−0.50	6.44**	−3.36*	−1.85	−7.95***
	No previous health condition	−7.02**	−2.69	−4.19*	4.33**	−1.99	−1.50	−4.82**

L1, Lockdown-1, 23 March–1 June 2020; L2, Lockdown-2, 2 November–2 December 2020; L3, Lockdown-3, 4 January–29 March 2021; NL1, Non-Lockdown-1, 1 June–November 2020; NL2, Non-Lockdown-2, 30 March 2021.

***P < 0.001, **P < 0.01, *P < 0.05.

In sum, comparing the period of Lockdown-1 with the period of eased restrictions immediately after, there was a decline in mental health symptoms across all sub-groups – except for individuals feeling lonely often, those aged 18–29 years old and those from a minority ethnic background (Table 4).

### Prevalence of elevated mental health symptoms during Lockdown-2

During Lockdown-2 the prevalence of reported mental health symptoms was at 30.72% (95% CI 28.02–33.41), a significant 21.78% increase from the first non-lockdown period preceding it. Demographic patterns were consistent with those observed in Lockdown-1 (Table 3). Women continued to report elevated mental health symptoms (36.99% [95% CI 33.44–40.55]) compared with men (24.21% [95% CI 20.54–27.88]), as did individuals aged 30–45 years (35.08% [95% CI 30.19–39.96]), followed closely by those aged 18–29 years (32.00% [95% CI 22.76–41.24]). There were no significant differences to report between different ethnic groups. Individuals reporting feeling lonely ‘often’ reported a greater prevalence of elevated mental health symptoms (84.09% [95% CI 75.91–92.27]), compared with those feeling lonely ‘sometimes’ (47.83% [95% CI 42.78–52.87]) or not at all (14.81% [95% CI 12.43–17.20]). Elevated mental health symptoms were higher for individuals working from home (33.58% [95% CI 30.05–37.12]) than those who did not (28.09% [95% CI 24.16–32.01]), and for those with long-term health conditions (35.06% [95% CI 31.3–38.89]) than those without (27.48% [95% CI 23.96–30.99]).

In sum, comparing the period of Lockdown-2 with the first period of eased restrictions (Table 4) showed an increase in elevated mental health symptoms for almost all population subgroups, with the exception of individuals who often felt lonely, and young (aged 18–29 years) as well as older adults (aged ≥60 years).

### Prevalence of elevated mental health symptoms during Lockdown-3 and subsequent lifting of restrictions

During England's final lockdown, the prevalence of elevated mental health symptoms was recorded at 29.10% (95% CI 26.96–31.23), a 22.58% significant decrease from Lockdown-2. Consistent with previous lockdowns (Tables 3), the prevalence of elevated mental health symptoms was higher for women (34.54% [95% CI 31.79–37.28]) than men (23.40% [95% CI 20.28–26.53]), for individuals aged 30–45 years (34.84% [95% CI 30.81-38.86]) than other age categories, and those feeling lonely often (82.96% [95% CI 76.92–89.01]) than lonely ‘sometimes' (43.75% [95% CI 39.97–47.54]) or not at all (12.88% [95% CI 11.28–14.47]), as well as individuals working from home (31.16% [95% CI 28.33–34.00]) than those who did not (26.980% [95% CI 23.79–30.19]), and finally, those reporting long-term health conditions (33.55% [95% CI 30.37–36.74]) than those not (25.63% [95% CI 22.87–28.40]). There were no significant differences to report between different ethnic groups.

Following the lifting of restrictions on 30 March 2021, the prevalence of reported mental health symptoms decreased to 22.53% (95% CI 20.41–24.64), a significant 22.58% decrease from the period encompassing Lockdown-3. This decline in reported mental health symptoms was observed across various population subgroups (Table 4). Notably, while women reported experiencing more elevated mental health symptoms (28.16% [95% CI 25.35–30.98]) compared with men (16.23% [95% CI 13.47–18.99]), men and women both experienced a similar decrease of about 30%. Individuals aged 18–29 years were the only age group that did not show a significant decline in elevated mental health symptoms from Lockdown-3 to the final easing of restrictions. No decline in reported mental health symptoms was seen for those feeling lonely ‘often’.

### Predictors of mental health

Table 5 presents the predictors of elevated mental health symptoms. Notably, being female, experiencing loneliness, having a long-term health condition and working from home were all identified predictors of elevated mental health symptoms. With the exception of the second lockdown period (which had no difference), all other lockdown and non-lockdown periods were less likely to report elevated levels of mental health symptoms. Age was not a significant predictor. In terms of ethnicity, compared with White individuals, Asian individuals were less likely to report poorer mental health, with other ethnicities not exhibiting a significant difference.

**Table 5:** Logistic regression model for the prevalence of elevated mental health symptoms across the pandemic (COVID-19 Survey Waves 1–9)

	Coefficients	Estimate	s.d.	*T*	Pr(>|*t*|) Significance
	(Intercept)	−2.381	0.172	−13.87	***
Gender (compared with Males)	Female	0.430	0.078	5.527	***
	Prefer not say	−1.425	1.091	−1.306	n/s
Work location (compared with Work at workplace)	Work at home	0.234	0.079	2.953	**
Age (compared with 16–29)	30–45	0.295	0.156	1.886	n/s
	46–59	0.238	0.148	1.609	n/s
	≥60	0.275	0.154	1.786	n/s
Ethnicity (compared with White)	Mix	−0.183	0.228	−0.801	n/s
	Asian	−0.337	0.170	−1.987	*
	Black	−0.370	0.316	−1.172	n/s
	Other	0.442	0.783	0.565	n/s
Loneliness (compared with never feel lonely)	Sometimes loneliness	1.674	0.078	21.567	***
	Often loneliness	3.572	0.168	21.204	***
Health condition (compared with not have health condition)	Have health condition	0.369	0.077	4.817	***
Time period (compared with first lockdown)	First non-lockdown	−0.358	0.069	−5.202	***
	Second lockdown	−0.104	0.092	−1.14	n/s
	Second non-lockdown	−0.480	0.079	−6.082	***
	Third lockdown	−0.276	0.069	−3.99	***

n/s, not significant.

***P < 0.001, **P < 0.01, *P < 0.05.

## Discussion

This study explored the impact of the COVID-19 pandemic and lockdowns on mental health in England before, during and after the pandemic. Our findings show a significant increase in the prevalence of self-reported elevated mental health symptoms during England's entire COVID-19 pandemic period, encompassing three national lockdowns, compared with the average of rates across England in the prior ten years (2009–2019). This study is unique in showing that an increase in elevated mental health symptoms during the COVID-19 pandemic occurred not only during and in the immediate aftermath of Lockdown-1, but over the subsequent year from March 2020 until March 2021.

Our study found no consistent significant difference in the rates of reported mental health symptoms across each of the three lockdowns, indicating that repeated lockdowns had enduring effects on mental health. Measures taken to control the pandemic therefore had a substantial and persistent impact on individual mental health. The rates of reported mental health symptoms within each of the three lockdowns in England, respectively, were significantly higher than the pre-pandemic average of rates across the prior 10 years. Moreover, there was a significant decline in reported mental health symptoms during periods of non-lockdown when restrictions were eased, indicating some improvements to population mental health. In this regard, our findings echo national polling data^34^ which during the first 6 months of the pandemic showed an increase in population mental health problems coinciding with periods of national lockdown and a decrease when restrictions were eased. When comparing periods of combined lockdown and non-lockdown, changes in the prevalence of reported mental health symptoms were greater for women, adults aged 30–45 years, those working from home and individuals feeling lonely ‘hardly ever’, as well as those with previous health conditions.

Taken together, findings from this study suggest that individuals were reactive to the effects of lockdown. However, as indicated above, this was not a uniform experience for all. The fact that reported rates of mental health symptoms were comparable across each of the three lockdowns, and higher than pre-pandemic levels, may be at odds with two past studies that found improvements in population mental health over repeated lockdowns;^17,18^ however, there are key differences which may explain the findings. A greater number of lockdowns, which were longer in duration and legally enforceable (versus ‘mild’ lockdowns with requests made for self-restraint in past studies^18^) operated in England, potentially indicating a more challenging psychological experience.

A comparison of the period between final eased restrictions at the end of Lockdown-3 up to May 2023 (2 years after lockdowns in England ended) also reveals elevated mental health symptoms compared with the pre-pandemic period. Caution should be exercised when interpreting these findings as attributable to the pandemic and/or lockdown in particular. While investigations continue exploring the ongoing effects of the pandemic on health and well-being,^35^ loneliness^25^ and healthcare provision,^36^ additional economic and political factors may reasonably be held accountable for the ongoing and profound levels of mental health symptoms.

### Impact on different sub-groups

The findings from this study suggest that the following were at risk for experiencing elevated mental health symptoms: being a woman, individuals aged between 30–45 and 46–59 years, those working from home, those feeling lonely and people with a long-term health condition, and lockdown periods. Further, our findings show that conditions of lockdown and non-lockdown affected the mental health of population subgroups differently. Similar to studies early in the pandemic,^2,4,5,18^ we found greater elevated levels of reported mental health symptoms under lockdown for women, people who worked from home, people with pre-existing health conditions, those aged 30–45 years and those who felt lonely ‘often’.

Previous studies have consistently highlighted the disproportionate effect of the pandemic on the mental health of women versus men.^2,3,5,14,17^ This effect may be explained by increased demands on caregiving^21,37^ – corroborating research arguing that despite increased full-time participation in the workforce, women still bear the greater burden for housework and child care responsibilities.^38^ Previous studies^2,17^ during the first lockdown have noted that younger, compared with older, adults experienced higher levels of depressive symptoms. We additionally found that individuals aged 30–45 years experienced elevated mental health symptoms which may indicate disruption to work,^22^ or explained by increased caregiving responsibilities^21^ or greater conformity of this age group to lockdown rules relative to young adults. Research notes the upending impact of the pandemic on workers, globally, with many facing teleworking for the first time,^39^ leading to disrupted work patterns and blurring of boundaries between work and family domains.^40^ Indeed, our findings show that mental health symptoms were consistently more elevated for homeworking individuals. For people with pre-existing health conditions, our findings corroborate extant research;^5,17^ lockdowns likely accelerated stress because of a loss of access to support, clinicians and positive activities. Existing data show increased levels of loneliness during the pandemic;^6,34^ however, less work has identified how mental health interacts with loneliness. Whereas some past research^34^ has collapsed categories of loneliness (i.e. feeling ‘lonely sometimes’ and feeling lonely ‘often’), the present study presents a more nuanced insight into loneliness by plotting these groups separately. Our findings show that the highest rates of elevated levels of mental health symptoms across all subgroups were experienced by people feeling lonely ‘often’ for whom there was no percentage change in reported elevated mental health symptoms from the final lockdown to when restrictions were finally eased. These individuals may have been unprepared for sustained periods of isolation, ill-equipped with coping strategies and potentially less willing or able to spend time on activities to alleviate loneliness, underscoring concerns of what some are referring to as the ‘loneliness pandemic’.^25^

Regarding risk factors for elevated mental health symptoms, while our regression analysis showed that compared with White individuals, Asian individuals were less likely to report mental health symptoms, with other ethnicities not exhibiting a significant difference, we note that the effect size for this calculation was low. No other differences on ethnicity were reported comparing across the different time periods. Evidence about the pandemic's impact on mental health by ethnicity is inconclusive, with some studies showing a disproportionate effect on ethnic minorities^2,20^ and some, no effect.^5^ Although there are clear differences between extant research which has focused on the UK, rather than individual nations as was the case here, our findings should be interpreted with caution. Ethnic minorities are underrepresented in the UKHLS^41^ (despite our best efforts in weighting the data). There was not enough statistical power to fully explore the mental health impact of the pandemic on the experiences of Black and minority ethnic individuals. We urge future research to focus on this area so that appropriate, targeted interventions can be put in place.

Perhaps one unique insight the present study adds to existing research on the mental health impact of the pandemic and lockdowns is that for those aged 18–29 years and those experiencing loneliness, there was no significant decline in rates of elevated mental health symptoms when final restrictions were eased on 30 March 2021. Our findings suggest that elevated mental health symptoms may have persisted for these subgroups beyond the official pandemic end.

### Study strengths and limitations

Drawing on longitudinal population-based data from before, during and after the pandemic, and thereby tracking changes in our sample over time, this study carries many advantages over studies relying on cross-sectional analysis with small convenience samples and those that have not conducted analysis on the prevalence of mental health both during and after lockdown periods. However, this study has several limitations.

The UKHLS is a high-quality probability sample cohort study which gathers data from individuals in private households, therefore excluding data from individuals in settings that were at particular risk from infection (i.e. prisons and nursing homes). The response rates in the COVID-19 survey are much lower than those in the UKHLS and therefore our findings may underestimate the true impact of mental health over each lockdown. Additionally, survey non-participation in both surveys may have introduced bias in our statistical estimates. Our analysis within England also did not account for regional differences in lockdowns^42^ which may have yielded differential patterns of elevated mental health symptoms across demographics. Both surveys also are underpowered in terms of their representation of ethnicity and older adults^41^ suggesting cautious interpretation of results. There are also limitations associated with our use of the GHQ-12, which is a widely used and valid tool for screening non-psychotic psychiatric cases in the population,^32^ but nonetheless remains a self-reported and non-clinical assessment of mental health, which may additionally underestimate ethnic disparities.^41^ It is also important to note that the elevated mental health symptoms do not reflect a clinical diagnosis.

Importantly, there is still much to research regarding factors (e.g. social support) that may curtail mental health symptoms, as well as intersectionality which may indicate that our findings may be more apparent in their association with other variables (e.g. ethnicity as it interacts with gender, or socioeconomic status).^43^

In conclusion a significant mitigation measure implemented during the COVID-19 pandemic was lockdowns. This study found an adverse mental health impact of repeated lockdowns in England compared with the average of rates from 10 years prior, with this impact not borne out uniformly across demographic subgroups. For future health emergencies, it is important that mental health services and interventions are aimed at reducing elevated mental health symptoms, potentially through coping strategies aimed at fostering adjustment under conditions of lockdown. This study's findings may help inform the development of such interventions. There is a need for ongoing research to identify mechanisms that explain the persistence in inequalities in mental health, in addition to specific support for vulnerable groups. Resources to make this possible need to be put in place by governments and policymakers.

## Supporting information

Dhensa-Kahlon et al. supplementary material Dhensa-Kahlon et al. supplementary material

## Data Availability

The data that support the findings of this study are openly available through the UK Data Service (https://www.understandingsociety.ac.uk/documentation/access-data/).

## Footnotes

1 As an example: stay-at-home orders in 2020 lasted for 92 days in England, 99 days in Wales, 68 in Scotland and 50 in Northern Ireland.
