# Supplementary material for: The mental health impact of repeated COVID-19 enforced lockdowns in England: evidence from the UK Household Longitudinal Study
Source: BJPsych Open. 2025 Jan 13;11(1):e16. doi: 10.1192/bjo.2024.803 (PMC11733484; doi:10.1192/bjo.2024.803)
Supplement: Dhensa-Kahlon et al. supplementary material [file S2056472424008032sup001.docx]

**Supplementary Materials**

**Table A: Study descriptive statistics**

|  |  | **Unweighted Population** | | | **Weighted Population** | | |
| --- | --- | --- | --- | --- | --- | --- | --- |
| **Wave*** | **Demographics** | **n** | **%** | **Prevalence of mental health symptoms %** | **n** | **%** | **Prevalence of mental health symptoms %** |
| **1** | **Overall** | **6093** | **81.10** | **34.19** | **7513** | **100** | **34.85** |
| 1 | Male | 2548 | 33.91 | 23.90 | 2147.29 | 28.58 | 25.15 |
| 1 | Female | 3541 | 47.13 | 41.6 | 2470.75 | 32.89 | 43.40 |
| 1 | Work Place | 1438 | 19.14 | 35.33 | 1278.07 | 17.01 | 34.79 |
| 1 | Work From Home | 1892 | 25.18 | 38.11 | 1327.12 | 17.66 | 39.07 |
| 1 | 18-29 | 356 | 4.74 | 48.31 | 465.23 | 6.19 | 41.40 |
| 1 | 30-45 | 1054 | 14.03 | 44.97 | 910.10 | 12.11 | 48.48 |
| 1 | 46-59 | 1711 | 22.77 | 36 | 1340.64 | 17.84 | 39.07 |
| 1 | 60+ | 2931 | 39.01 | 27.50 | 1980.39 | 26.36 | 27.45 |
| 1 | White | 5505 | 73.27 | 33.77 | 4374.54 | 58.23 | 34.70 |
| 1 | Mixed | 85 | 1.13 | 45.88 | 43.15 | 0.57 | 46.43 |
| 1 | Asian | 309 | 4.11 | 37.22 | 123.45 | 1.64 | 39.86 |
| 1 | Black | 89 | 1.18 | 34.83 | 45.81 | 0.61 | 19.62 |
| 1 | Other | 22 | 0.29 | 31.82 | 18.89 | 0.25 | 28.87 |
| 1 | Hardly | 4129 | 54.96 | 20.59 | 3004.56 | 39.99 | 19.71 |
| 1 | Sometimes | 1605 | 21.36 | 57.45 | 1278.63 | 17.02 | 57.58 |
| 1 | Often | 359 | 4.78 | 86.63 | 346.68 | 4.61 | 82.24 |
| **2** | **Overall** | **6093** | **81.10** | **29.23** | **7513** | **100** | **31.33** |
| 2 | Male | 2552 | 33.97 | 21 | 2218.79 | 29.53 | 23.63 |
| 2 | Female | 3541 | 47.13 | 35.16 | 2464.50 | 32.80 | 38.27 |
| 2 | Work Place | 1451 | 19.31 | 29.15 | 1358.91 | 18.09 | 30.10 |
| 2 | Work From Home | 1882 | 25.05 | 33.53 | 1381.04 | 18.38 | 34.91 |
| 2 | 18-29 | 352 | 4.69 | 39.20 | 597.76 | 7.96 | 35.51 |
| 2 | 30-45 | 1049 | 13.96 | 38.23 | 959.25 | 12.77 | 40.98 |
| 2 | 46-59 | 1703 | 22.67 | 29.83 | 1343.83 | 17.89 | 34.91 |
| 2 | 60+ | 2949 | 39.25 | 24.35 | 1849.27 | 24.61 | 25.62 |
| 2 | White | 5505 | 73.27 | 28.92 | 4372.71 | 58.20 | 31.01 |
| 2 | Mixed | 85 | 1.13 | 42.35 | 48.36 | 0.64 | 44.04 |
| 2 | Asian | 309 | 4.11 | 29.13 | 158.6 | 2.11 | 35.22 |
| 2 | Black | 89 | 1.18 | 28.09 | 52.52 | 0.70 | 26.34 |
| 2 | Other | 22 | 0.29 | 18.18 | 21.14 | 0.28 | 29.55 |
| 2 | Hardly | 4163 | 55.41 | 15.11 | 3010.24 | 40.07 | 14.97 |
| 2 | Sometimes | 1611 | 21.44 | 54.69 | 1335.81 | 17.78 | 55.51 |
| 2 | Often | 319 | 4.25 | 84.95 | 337.24 | 4.49 | 81.62 |
| 2 | No Previous Health Condition | 2852 | 37.96 | 27.28 | 2268.81 | 30.2 | 27.94 |
| 2 | Previous Health Condition | 3241 | 43.14 | 30.95 | 2414.48 | 32.14 | 34.52 |
| **3** | **Overall** | **6093** | **81.10** | **26.69** | **7513** | **100** | **28.84** |
| 3 | Male | 2547 | 33.90 | 19.71 | 2194.46 | 29.21 | 23.70 |
| 3 | Female | 3542 | 47.14 | 31.73 | 2435.46 | 32.42 | 33.69 |
| 3 | Work Place | 1485 | 19.77 | 26.53 | 1423.91 | 18.95 | 28.02 |
| 3 | Work From Home | 1843 | 24.53 | 30.39 | 1307 | 17.40 | 31.82 |
| 3 | 18-29 | 351 | 4.67 | 36.18 | 606.36 | 8.07 | 35.36 |
| 3 | 30-45 | 1047 | 13.94 | 34.67 | 991.41 | 13.20 | 37.69 |
| 3 | 46-59 | 1698 | 22.60 | 27.56 | 1334.95 | 17.77 | 31.82 |
| 3 | 60+ | 2960 | 39.40 | 21.99 | 1789.9 | 23.82 | 22.58 |
| 3 | White | 5505 | 73.27 | 26.16 | 4327.19 | 57.60 | 28.05 |
| 3 | Mixed | 85 | 1.13 | 40 | 49.58 | 0.66 | 52.63 |
| 3 | Asian | 309 | 4.11 | 29.45 | 166.26 | 2.21 | 37.36 |
| 3 | Black | 89 | 1.18 | 28.09 | 54.40 | 0.72 | 37.45 |
| 3 | Other | 22 | 0.29 | 18.18 | 20.64 | 0.27 | 28.97 |
| 3 | Hardly | 4212 | 56.06 | 13.25 | 2964.34 | 39.46 | 13.18 |
| 3 | Sometimes | 1612 | 21.46 | 51.99 | 1363.40 | 18.15 | 50.28 |
| 3 | Often | 267 | 3.55 | 86.14 | 318.38 | 4.24 | 83.01 |
| 3 | No Previous Health Condition | 2746 | 36.55 | 24.76 | 2196.65 | 29.24 | 25.21 |
| 3 | Previous Health Condition | 3347 | 44.55 | 28.26 | 2450.93 | 32.62 | 32.10 |
| **4** | **Overall** | **6093** | **81.10** | **22.55** | **7513** | **100** | **24.30** |
| 4 | Male | 2549 | 33.93 | 16.79 | 2195.15 | 29.22 | 19.89 |
| 4 | Female | 3542 | 47.14 | 26.71 | 2456.74 | 32.70 | 28.25 |
| 4 | Work Place | 1477 | 19.66 | 22.88 | 1365.64 | 18.18 | 23.36 |
| 4 | Work From Home | 1838 | 24.46 | 25.14 | 1348.30 | 17.95 | 26.5 |
| 4 | 18-29 | 346 | 4.61 | 27.46 | 599.71 | 7.98 | 27.56 |
| 4 | 30-45 | 1046 | 13.92 | 28.39 | 990.23 | 13.18 | 31.54 |
| 4 | 46-59 | 1692 | 22.52 | 24.88 | 1346.03 | 17.92 | 26.50 |
| 4 | 60+ | 2972 | 39.56 | 18.47 | 1790.44 | 23.83 | 18.26 |
| 4 | White | 5505 | 73.27 | 22.23 | 4354.34 | 57.96 | 23.96 |
| 4 | Mixed | 85 | 1.13 | 30.59 | 47.82 | 0.64 | 36.75 |
| 4 | Asian | 309 | 4.11 | 24.92 | 151.59 | 2.02 | 26.46 |
| 4 | Black | 89 | 1.18 | 21.35 | 48.84 | 0.65 | 26.99 |
| 4 | Other | 22 | 0.29 | 22.73 | 21.42 | 0.29 | 30.63 |
| 4 | Hardly | 4167 | 55.46 | 10.10 | 3000.58 | 39.94 | 9.38 |
| 4 | Sometimes | 1671 | 22.24 | 43.81 | 1362.41 | 18.13 | 44.43 |
| 4 | Often | 255 | 3.39 | 86.67 | 289.48 | 3.85 | 84.23 |
| 4 | No Previous Health Condition | 2711 | 36.08 | 20.14 | 2176.34 | 28.97 | 20.99 |
| 4 | Previous Health Condition | 3382 | 45.02 | 24.48 | 2476.13 | 32.96 | 27.21 |
| **5** | **Overall** | **6093** | **81.10** | **23.45** | **7513** | **100** | **24.59** |
| 5 | Male | 2547 | 33.90 | 17.08 | 2232.84 | 29.72 | 17.86 |
| 5 | Female | 3540 | 47.12 | 28.05 | 2489.21 | 33.13 | 30.64 |
| 5 | Work Place | 1605 | 21.36 | 24.86 | 1542.52 | 20.53 | 24.70 |
| 5 | Work From Home | 1695 | 22.56 | 24.42 | 1267.23 | 16.87 | 26.29 |
| 5 | 18-29 | 344 | 4.58 | 27.91 | 683.62 | 9.10 | 27.46 |
| 5 | 30-45 | 1034 | 13.76 | 28.92 | 1013.48 | 13.49 | 30.04 |
| 5 | 46-59 | 1691 | 22.51 | 24.96 | 1349.15 | 17.96 | 26.29 |
| 5 | 60+ | 2990 | 39.80 | 19.93 | 1759.36 | 23.42 | 19.76 |
| 5 | White | 5505 | 73.27 | 23.29 | 4385.93 | 58.38 | 24.50 |
| 5 | Mixed | 85 | 1.13 | 25.88 | 50.67 | 0.67 | 24.68 |
| 5 | Asian | 309 | 4.11 | 22.65 | 177.77 | 2.37 | 28.73 |
| 5 | Black | 89 | 1.18 | 24.72 | 57.71 | 0.77 | 20.75 |
| 5 | Other | 22 | 0.29 | 18.18 | 22.02 | 0.29 | 27.08 |
| 5 | Hardly | 4206 | 55.98 | 11.44 | 2997.66 | 39.90 | 11.04 |
| 5 | Sometimes | 1599 | 21.28 | 44.90 | 1442.08 | 19.19 | 42.06 |
| 5 | Often | 287 | 3.82 | 80.14 | 289.68 | 3.86 | 77.80 |
| 5 | No Previous Health Condition | 2681 | 35.68 | 20.59 | 2243.12 | 29.86 | 21.77 |
| 5 | Previous Health Condition | 3412 | 45.41 | 25.70 | 2486.58 | 33.10 | 27.13 |
| **6** | **Overall** | **6093** | **81.10** | **28.92** | **7513** | **100** | **29.83** |
| 6 | Male | 2546 | 33.89 | 22.43 | 2242.32 | 29.85 | 23.75 |
| 6 | Female | 3542 | 47.14 | 33.60 | 2518 | 33.52 | 35.44 |
| 6 | Work Place | 1516 | 20.18 | 27.97 | 1501.30 | 19.98 | 28.03 |
| 6 | Work From Home | 1773 | 23.60 | 31.53 | 1382.86 | 18.41 | 33.29 |
| 6 | 18-29 | 336 | 4.47 | 37.20 | 682.32 | 9.08 | 33.29 |
| 6 | 30-45 | 1032 | 13.74 | 33.72 | 1049.30 | 13.97 | 37.29 |
| 6 | 46-59 | 1681 | 22.37 | 31.47 | 1354.04 | 18.02 | 33.29 |
| 6 | 60+ | 3013 | 40.10 | 24.79 | 1772.55 | 23.59 | 23.70 |
| 6 | White | 5505 | 73.27 | 28.72 | 4431.75 | 58.99 | 29.70 |
| 6 | Mixed | 85 | 1.13 | 35.29 | 51.07 | 0.68 | 39.75 |
| 6 | Asian | 309 | 4.11 | 27.83 | 188.28 | 2.51 | 29.27 |
| 6 | Black | 89 | 1.18 | 31.46 | 56.98 | 0.76 | 30.27 |
| 6 | Other | 22 | 0.29 | 22.73 | 22.12 | 0.29 | 31.24 |
| 6 | Hardly | 3867 | 51.47 | 14.58 | 2860.82 | 38.08 | 13.78 |
| 6 | Sometimes | 1900 | 25.29 | 48.26 | 1580.11 | 21.03 | 46.82 |
| 6 | Often | 323 | 4.30 | 86.69 | 340.64 | 4.53 | 85.80 |
| 6 | No Previous Health Condition | 2632 | 35.03 | 26.44 | 2233.60 | 29.73 | 27.63 |
| 6 | Previous Health Condition | 3461 | 46.07 | 30.80 | 2548.4 | 33.92 | 31.76 |
| **7** | **Overall** | **6093** | **81.10** | **31.22** | **7513** | **100** | **31.74** |
| 7 | Male | 2547 | 33.90 | 24.19 | 2276.46 | 30.30 | 24.41 |
| 7 | Female | 3541 | 47.13 | 36.23 | 2491.21 | 33.16 | 38.47 |
| 7 | Work Place | 1371 | 18.25 | 30.93 | 1408.39 | 18.75 | 29.89 |
| 7 | Work From Home | 1881 | 25.04 | 34.18 | 1476.95 | 19.66 | 34.83 |
| 7 | 18-29 | 335 | 4.46 | 39.10 | 662.62 | 8.82 | 29.90 |
| 7 | 30-45 | 1024 | 13.63 | 39.26 | 1072.58 | 14.28 | 41.20 |
| 7 | 46-59 | 1665 | 22.16 | 31.23 | 1348.01 | 17.94 | 34.83 |
| 7 | 60+ | 3042 | 40.49 | 27.55 | 1765.77 | 23.50 | 27.44 |
| 7 | White | 5505 | 73.27 | 31.17 | 4413.68 | 58.75 | 31.68 |
| 7 | Mixed | 85 | 1.13 | 40 | 52.89 | 0.70 | 44.16 |
| 7 | Asian | 309 | 4.11 | 29.13 | 189.79 | 2.53 | 27.69 |
| 7 | Black | 89 | 1.18 | 26.97 | 59.74 | 0.80 | 33.03 |
| 7 | Other | 22 | 0.29 | 22.73 | 23.52 | 0.31 | 30.78 |
| 7 | Hardly | 3712 | 49.41 | 15.14 | 2705.08 | 36.01 | 13.66 |
| 7 | Sometimes | 1985 | 26.42 | 50.23 | 1617.43 | 21.53 | 47.14 |
| 7 | Often | 395 | 5.26 | 86.84 | 451.49 | 6.010 | 84.93 |
| 7 | No Previous Health Condition | 2579 | 34.33 | 28.73 | 2181.92 | 29.04 | 28.79 |
| 7 | Previous Health Condition | 3514 | 46.77 | 33.04 | 2592.35 | 34.50 | 34.24 |
| **8** | **Overall** | **6093** | **81.10** | **25.46** | **7513** | **100** | **25.48** |
| 8 | Male | 2549 | 33.93 | 19.93 | 2214.77 | 29.48 | 20.73 |
| 8 | Female | 3541 | 47.13 | 29.40 | 2467.59 | 32.84 | 29.74 |
| 8 | Work Place | 1450 | 19.30 | 24.62 | 1374.27 | 18.29 | 24.56 |
| 8 | Work From Home | 1813 | 24.13 | 25.70 | 1372.10 | 18.26 | 26.65 |
| 8 | 18-29 | 333 | 4.43 | 32.13 | 576.16 | 7.67 | 27.98 |
| 8 | 30-45 | 1025 | 13.64 | 29.56 | 973.06 | 12.95 | 30.24 |
| 8 | 46-59 | 1646 | 21.91 | 25.70 | 1308.42 | 17.42 | 26.65 |
| 8 | 60+ | 3066 | 40.81 | 23.16 | 1890.18 | 25.16 | 22.15 |
| 8 | White | 5505 | 73.27 | 25.45 | 4378.97 | 58.29 | 25.48 |
| 8 | Mixed | 85 | 1.13 | 34.12 | 49.53 | 0.66 | 41.62 |
| 8 | Asian | 309 | 4.11 | 21.68 | 159.61 | 2.12 | 18.15 |
| 8 | Black | 89 | 1.18 | 23.60 | 52.57 | 0.70 | 23.83 |
| 8 | Other | 22 | 0.29 | 18.18 | 21.62 | 0.29 | 31.66 |
| 8 | Hardly | 3934 | 52.36 | 12.28 | 2826.16 | 37.62 | 11.08 |
| 8 | Sometimes | 1860 | 24.76 | 44.52 | 1568.68 | 20.88 | 41.29 |
| 8 | Often | 299 | 3.98 | 80.27 | 293.67 | 3.91 | 79.63 |
| 8 | No Previous Health Condition | 2540 | 33.81 | 21.38 | 2056.19 | 27.37 | 21.61 |
| 8 | Previous Health Condition | 3553 | 47.29 | 28.37 | 2632.33 | 35.04 | 28.51 |
| **9** | **Overall** | **6093** | **81.10** | **21.24** | **7513** | **100** | **22.05** |
| 9 | Male | 2549 | 33.93 | 15.89 | 2669.26 | 35.53 | 16.35 |
| 9 | Female | 3541 | 47.13 | 25.05 | 3048.62 | 40.58 | 27.01 |
| 9 | Work Place | 1612 | 21.46 | 20.60 | 1848.40 | 24.60 | 21.81 |
| 9 | Work From Home | 1646 | 21.91 | 23.45 | 1462.22 | 19.46 | 23.86 |
| 9 | 18-29 | 316 | 4.21 | 28.80 | 727.91 | 9.69 | 23.16 |
| 9 | 30-45 | 1008 | 13.42 | 28.08 | 1139.34 | 15.16 | 27.69 |
| 9 | 46-59 | 1606 | 21.38 | 22.10 | 1554.67 | 20.69 | 23.86 |
| 9 | 60+ | 3151 | 41.94 | 17.71 | 2342.68 | 31.18 | 18.10 |
| 9 | White | 5547 | 73.83 | 21.06 | 5375.98 | 71.56 | 21.98 |
| 9 | Mixed | 87 | 1.16 | 29.89 | 60.21 | 0.80 | 27.91 |
| 9 | Asian | 318 | 4.23 | 21.70 | 162.70 | 2.17 | 20.96 |
| 9 | Black | 91 | 1.21 | 17.58 | 50.67 | 0.67 | 21.69 |
| 9 | Other | 22 | 0.29 | 18.18 | 18.26 | 0.24 | 4.98 |
| 9 | Hardly | 4310 | 57.37 | 10.16 | 3784.89 | 50.38 | 9.89 |
| 9 | Sometimes | 1533 | 20.40 | 41.68 | 1638.78 | 21.81 | 37.92 |
| 9 | Often | 250 | 3.33 | 86.80 | 299.71 | 3.99 | 88.76 |
| 9 | No Previous Health Condition | 2506 | 33.36 | 17.60 | 2536.18 | 33.76 | 17.22 |
| 9 | Previous Health Condition | 3587 | 47.74 | 23.78 | 3187.20 | 42.42 | 25.89 |

*Covid-19 Survey Waves
